# Morbus Coxarius

**Published:** 1878-04

**Authors:** Edwin R. Barnes


BUFFALO

### Medical and Surgical Journal.

VOL. XVII. APRIL, 1878. No. 9.

### Original Communications.

———:o:———

ART I.—*Morbus Coxarius* By EDWIN R. BARNES, M. D. Read before the Buffalo Medical Association, March 5th, 1878.

Within comparatively recent years much discussion, research and experiment have been devoted to the nature, causation and mode of treatment of the subject designated by the above title, and the result has been rejection, by the later teachers, of a theory which had long been established as unquestionable.—

That theory was that the strumous diathesis, or cachexia is the originating source of this disease, and the element to which is due its susceptive phenomena. Paget says of scrofula: "It is a state of constitution distinguished in some measure by peculiarities of appearance even during health, but much more by peculiar liability to certain diseases, including pulmonary phthisis. The chief of these 'scrofulous' diseases are various swellings of the lymphatic glands. * * * But besides these it is usual to reckon as scrofulous certain inflammations of the joints; slowly progressive 'carious' ulcerations of bones," etc. It was to this indefinite and not hopeful domain of struma that the causation of "morbus coxarius" was referred, and as a consequence the *local* treatment evolved from such a theory was insufficient and inadequate, and the great effort was expended in the hoveless attempt to overcome the constitutional defect. We know how unsatisfactory were the results obtained.

I shall not attempt to review here the arguments against this theory, but simply state that the cause of this disease is now considered to be generally, if not exclusively, traumatic, and that while the disease may be developed in a child of scrofulous habit, even more readily under the same circumstances than in a healthy subject, yet it is much less apt to occur in a strumous than in a robust subject, because the more vigorous habit of the latter will be much more liable to expose it to a traumatic cause. The strumous theory, as the chief predisposing cause of the disease, having been discarded by the later writers, the local management of the lesion has assumed its proper place, and with modern surgical appliances we have been enabled to more successfully combat this previously unmanageable complaint. Erichsen says that "morbus coxarius" "is usually considered a distinct affection apart from other joint diseases," "having so many points of peculiar and serious importance," but the views which have been established as to "morbus coxarius" have likewise rescued from other joint diseases the incubus of a hopeless theory.

One evidence against the strumous or constitutional theory which it seems difficult to answer, is that a patient in an advanced stage of the disease, with general symptoms which would indicate a speedy termination of life, may, if the cause is removed, say by the exsection of the head of the femur, entirely regain his general health; a condition not to be expected from a constitutional causation.

I remember hearing Professor Markoe state many years ago, that it was his experience, that if a case of "morbus coxarius" was recovered from, it was apt to be the *last manifestation* of struma to which the subject would give expression.

Sayre remarks under this head, "Another fact worthy of consideration is that a very large proportion of cases of the disease occur in children, while the scrofulous condition is by no means so restricted. If, therefore, we still adhere to the scrofulous theory, we are forced to conclude that the diathesis, which in childhood develops itself in joint disease, manifests itself in some other way after puberty. This I cannot believe." He is convinced that the cachectic condition so often seen is the result and not the cause of the disease.

Passing from this view of the subject and considering the disease as generally purely local in origin, I proceed to a brief reference to the anatomy of the parts most directly involved in the disease. These are, generally speaking, 1st, the os-innominatum and os-femoris, more closely, the acetabulum and the head of the femur—the latter being received into the former by an enarthrodial articulation which permits more or less movement in all directions. These latter, i. e. the os femoris and the acetabulum are cancellous in structure, are covered by a thin lamella of compact tissue, and are quite vascular and subject to inflammation. 2nd. An articular cartilage, which covers the head of the femur and the acetabulum, except at the pit called fundus acetabuli which is lined or cushioned with fat, and the depression of the head of the femur for the insertion of the ligamentum teres. 3d. The cotyloid ligament, a fibro cartilaginous rim attached to the margin of the acetabulum. 4th. The fibrinous transverse ligament, which connects the edges of the acetabular notch and forms of it a foramen for the transmission of nutrient vessels and nerves. 5th. The ligamentum teres, which is described as fibrous tissue attached to the depression in the head of the femur, and to the margins of the acetabular notch and becoming continuous with the transverse ligament, but which is also composed largely of areolar tissue and fat "encompassing the vessels and nerves passing to and from the head of the femur." 6th. The capsular ligament, reinforced by the powerful illio femoral ligament which I shall mention without description. 7th. The synovial membrane, lining the capsular ligament, reflected upon and covering the acetabulum, the ligamentum teres and part of the neck of the femur. 8th. The periosteum, limited in its approach to the joint structures by the capsular ligament with which it is supposed by some authorities to be continuous and similar. 9th. The muscles relating to this articulation, which I shall not describe further than to say that they control its movements and that their action when contracted is to cause the head to impinge more firmly upon its point of resistance, the acetabulum, and *often to fix the head more or less in one position.* 10th. The epiphyseal cartilage, which before adolescence, joins the head and neck of the femur, but separates the nutrient supply of the one from that of the other. 11th. The branches of the obturator artery and nerve, which, through the ligamentum teres, nourish the imperfectly joined head of the femur.

So much for the anatomy of the joint.

It may be said that all or nearly all of these parts become directly or indirectly implicated in the disease as it progresses, and contribute to make up the *tout ensemble* of symptoms and morbid phenomena which present themselves in the different stages of its development. At whatever point *originating*, other parts soon become involved, so that it is difficult (and not practically important as regards treatment,) to decide which part becomes first affected. The authorities which I have consulted however have indicated the following four structures, any one of which may be primarily affected, viz: 1st, the periosteum, 2nd, the synovial membrane, 3d, the ligamentum teres, and 4th, the vascular supply, permeating the superficial lamellæ of the head of the femur and of the acetabulum and underlying the cartilages thereof.

In regard to the first structure, the periosteum, this is not directly connected with the joint elements involved in this disease yet it seems to be recognized as being sometimes the starting point in the series of symptoms which ensue. It is superficial over the great trochanter, liable to exposure, to changes of temperature and to blows, which may cause periostitis, hyperæmia, impaired nutrition of structures beneath it, subperiostial suppuration, and transmission of the disease to other structures.

The *synovial membrane* seems to have a considerable degree of susceptibility to morbid action, and may be deemed a seat of the primary lesion, inflammation in this membrane, though developed by other causes, is most generally produced by exposure to sudden changes of temperature, after violent exercise, this inflammation is always followed by effusion, and may be sub-acute and comparatively trivial, or very acute and tending rapidly to destructive action, and the complication of other structures.

The *ligamentum teres* presents considerable claim to the first rank as a primary lesion. From its composition it must be considered as highly susceptible to morbid action. It is subject to contusion from violence to the great trochanter, when the thigh is in position of adduction and eversion, also to violent straining from extreme motions of the joint which may produce partial or complete rupture, and separation from its attachments to bones, hence may result necrosis of the femur and the involvement of cartilaginous and other structures.

In respect to the *vascular supply in the articular lamella*, this is subject to concussion from jumping and other causes, producing rupture of some of the vessels which are situated in the bone just beneath the articular cartilage and causing an extravasation, which at first may seem slight and not very noticeable, but being subjected to continuous irritation will ultimately give rise to serious complications.

It is from one of these four sources, so far as I can learn, that the disease originates.

I now hasten on to the symptoms.—These are classified under the *three stages*, the 1st, 2nd and 3d, into which the disease is divided in accordance with its different degrees of progress. A brief descriptive definition of these stages is quite suggestive. Bauer describes the first stage as one of congestive hyperæmia; Sayre, as the stage of irritation and limited motion. The second stage is characterized as that of effusion, with apparent lengthening of the limb. The third stage, as that of ruptured capsule, with apparent shortening.

The symptoms of the first stage are often obscure, or difficult to detect with certainty, but their recognition is important, because at this stage the disease may be arrested, and all serious complications avoided. They are briefly, tenderness and pain, slight abduction and slight flexion at the knee and hip, but no eversion of the limb, a certain amount of muscular rigidity particularly of the psoas magnus, illiacus and adductus which limits attempts at adduction, abduction to a less degree, flexion and extension, and finally, atrophy of the thigh and entire limb, and perhaps, some swelling about the joint. Such, briefly are the symptoms of the first stage; I shall attempt details as to methods of examination, but only say that *tenderness* may be recognized by manipulations about the joint, by forcibly or suddenly compressing the joint surfaces, by sweeping movements of the joint, and by observing that the patient standing naked before the surgeon, habitually throws the weight of the body upon the sound limb, causing the relaxation of the muscles on the affected side, the flattening of the natis and the lowering of the gluteo-femoral crease and rendering it more shallow.

*Muscular rigidity* may be observed by attempting movements of the limb with the patient reclining naked upon a smooth and unyielding surface. *Atrophy* by actual measurement. Bauer says, "The symptom of attenuation is co-ordinate with that of muscular contraction and never observed without the latter." He attributes this symptom to impeded innervation and impeded nutrition. The diminished mobility of the joint no doubt aids to produce atrophy.

As to *pain*, this may or may not be experienced in the first stage, except by motion or compression of the joint surfaces. This pain may be referred to the hip joint, or to the knee, when the disease manifests itself immediately after an injury, and the pain is immediate, constant and intense. Dr. Sayre regards the disease as due probably either to synovitis or periostitis of the great trochanter. When the pain is not developed until late in the first, or not until the second stage, he thinks the seat of the disease is in the articular lamella. In reference to the pain being referred to the knee, I would remark that in joint diseases, (as elsewhere) we recognize two kinds of pain, structural and reflex, the former emanating directly from the diseased structure, the latter proceeding in a circuitous manner from the spinal cord, and manifesting itself in parts not directly connected with the affected articulation.

The morbid impression being excited by local disturbance, is conveyed to the spinal cord, thence backward to the muscles appertaining to the affected joint, or to the next articulation as in coxalgia. The latter mode is rather an exception, and is explained thus: "that the same nerve (the obturator) supplies both joints with sensitive fibres."

It would be interesting, did time and my opportunities permit, to study more fully this subject of pain and joint disease, its influence on the actual progress of the disease, its influence in causing, in a more advanced stage, spasms, permanent rigidity or so-called contraction and structural change of muscle,—but I pass to the symptoms of the second stage. They are the same as those of the first but increase in degree. We have, as before pain, tenderness, flattening of the gluteal region, sinking of the gluteal fold, abduction, flexion, muscular rigidity and atrophy, but *more marked*, also apparent lengthening. We have in addition, *eversion* of the limb, intense reflex pains not referred to the knee, occurring mostly at night, and often permanent structural change in muscles, termed contraction. These are peculiar to this stage except contraction, which continues into the next stage. These reflex pains, at night, and in sleep, are considered by Bauer to prove that the trophic, or ganglionic province is principally, if not exclusively involved. Sayre more simply describes it as due to the relaxation of muscles during sleep, the movement of the joint surfaces causing the excitation of reflex action which stimulates to sudden muscular contraction and compression or concussion of the articular surfaces. During the day muscular contraction is more constant. Both the sudden concussion and the constant contraction aggravate the destructive joint changes by pressure.

The eversion marked in the second stage, is caused by effusion within the joint, which "forces the movable part of the joint into certain positions denoting the greatest capacity of the articulation." The experiments of Weber and Bonnet showed, by injecting fluid into the hip joint in the dead subject, that the result was always the production of eversion, flexion and abduction of the thigh and immobility of the joint. The struggle between the contraction of the adductors of the limb and the distended capsule increases pain and aggravates disease.

The symptoms of the *third stage* present a marked change, nearly the reverse of those of the second. We have now inversion instead of eversion, adduction instead of abduction, flexion at the hip, but perhaps not at the knee, elevation of the pelvis upon the affected side and the projecting backward of the natis instead of the reverse, and there is comparative freedom from pain. These changes are caused by rupture of the capsule, and effusion from it into surrounding tissues where openings may be made. The action of the adductors is now unopposed and they cause the adduction and inversion. The perforation of the acetabulum by the head of the femur, or the presence of osteophytes may fix the limb and prevent the changes described. Actual shortening now generally exists from loss of substance in the head of the femur and the acetabulum. Spontaneous luxation very seldom occurs though there may be much change of position from such loss of substance. Traumatic dislocation may be easily produced. Caries and destruction of cartilages occur in this stage and may have existed in the second stage.

Time will not permit me to enter on the subject of treatment which may be left to verbal discussion. I will only say that rest and position, extension, the relief of compression of the joint surfaces while permitting motion, are the points aimed at by surgical appliances.

The principal operative procedures employed, are myotomy or tenotomy, the relief of effusion within the capsule by puncture or by aspiration, and exsection of the hip joint.

